# Possibilities of Automated Diagnostics of Odontogenic Sinusitis According to the Computer Tomography Data

**DOI:** 10.3390/s21041198

**Published:** 2021-02-08

**Authors:** Oleg G. Avrunin, Yana V. Nosova, Ibrahim Younouss Abdelhamid, Sergii V. Pavlov, Natalia O. Shushliapina, Waldemar Wójcik, Piotr Kisała, Aliya Kalizhanova

**Affiliations:** 1 Department of Biomedical Engineering, Faculty of Electronic and Biomedical Engineering Kharkiv National University of Radio Electronics, 61166 Kharkiv, Ukraine; oleh.avrunin@nure.ua (O.G.A.); yana.nosova@nure.ua (Y.V.N.); info@nure.ua (I.Y.A.); 2 Department of Biomedical Engineering, Vinnytsia National Technical University, 21021 Vinnytsia, Ukraine; psv@vntu.edu.ua; 3 Department of Otorhinolaryngology, Stomatological Faculty Kharkiv National Medical University, 61022 Kharkiv, Ukraine; meduniver@knmu.kharkov.ua; 4 Institute of Electronic and Information Technologies, Faculty Electrical Engineering and Computer Science, Lublin University of Technology, 20-618 Lublin, Poland; waldemar.wojcik@pollub.pl; 5 Institute of Information and Computational Technologies CS MES RK, 050010 Almaty, Kazakhstan; kalizhanova_aliya@mail.ru; 6 University of Power Engineering and Telecommunications, 050013 Almaty, Kazakhstan

**Keywords:** bone densitography, discriminant analysis, pathology diagnosis, sinusitis

## Abstract

Individual anatomical features of the paranasal sinuses and dentoalveolar system, the complexity of physiological and pathophysiological processes in this area, and the absence of actual standards of the norm and typical pathologies lead to the fact that differential diagnosis and assessment of the severity of the course of odontogenic sinusitis significantly depend on the measurement methods of significant indicators and have significant variability. Therefore, an urgent task is to expand the diagnostic capabilities of existing research methods, study the significance of the measured indicators, and substantiate the expediency of their use in the diagnosis of specific pathologies in an automated mode. Methods of digital filtering, image segmentation and analysis, fluid dynamics, and statistical and discriminant analysis were used. Preliminary differential diagnosis of odontogenic sinusitis can be performed by densitemetric analysis of tomographic images of the maxillary sinuses, performed using frontal multiplanar reconstructions according to a given algorithm. The very manifestation of the characteristic changes in the densitography of the maxillary sinus allows for the initiation of certain pathological processes and permits the development of the effectiveness of the diagnosis of the pathology of the sinus sinuses, which can be realized automatically in real life.

## 1. Introduction

The problem of diagnosis, treatment, rehabilitation, and, especially, prevention of odontogenic sinusitis continues to remain relevant at the present time, since the bulk of patients are young and middle-aged people, that is, the working-age population [1]. In recent years, there has been a steady upward trend in the number of patients with this pathology. Today in the world, there are numerous methods for the diagnosis and treatment of odontogenic maxillary sinusitis, which are widely used as surgical and conservative options for medical care [1,2,3]. However, despite significant advances in diagnosis and progress in the treatment of this category of patients, most clinicians report a large number of recurrences and complications after surgery, ranging from 30% to 50% [3,4]. In most cases, these issues are associated with difficulties in diagnosis, specific anatomical and physiological properties of the maxillary sinuses, polyetiological factors of the disease, and numerous clinical manifestations, which in turn are associated with complex treatment and high risk of recurrence and complications. One of the reasons for failures in the treatment of odontogenic maxillary sinusitis is the human factor, so the topical issue is the use of automated processing and intelligent analysis of diagnostic data in predicting the course of the disease and correction of treatment approaches [5,6,7,8].

The use of such systems allows specialists to make a diagnosis with high reliability, provide advice on the choice of treatment tactics, predict the course of the disease, determine the level of threat, and prevent various complications. It should be noted that such automated systems have an additional, advisory function in improving the quality of qualified care, and the main responsibility for making final decisions rests with the physician. The occurrence of odontogenic sinusitis-inflammation of the walls of the maxillary sinus is associated with the spread of infection from diseased teeth, which is facilitated by anatomical features in the form of close adherence of the floor of the maxillary sinus to the apex of the roots of the teeth. A common cause of the development of the disease is errors in endodontic treatment of teeth and dental implantation, carrying out instruments for processing root canals (root needles, drillers, canal fillers, pulpextractors) as well as filling material and an implant for the apex of the tooth root into the sinus cavity [1,2,3]. Less commonly, fragments of tooth roots are foreign bodies in the sinus cavity. It is also possible to infect the sinus during surgery with perforation of the bottom of the maxillary sinus cavity, for example, when extracting the first and second molars of the upper jaw, when resecting the root apex, removing impacted teeth, sequestrectomy, and replanting a dental implant. Typical complaints of patients with acute maxillary sinusitis were difficulty in nasal breathing, rhinorrhea, loss of smell, head and facial pain, subfebrile fever, as well as nocturnal cough and sleep disturbance.

Odontogenic sinusitis (in contrast to rhinogenic sinusitis) has the following distinctive features: isolated lesion of one of the maxillary sinuses, pain in the tooth or in periodontal tissues, preceding the disease, disruption of the face configuration as a result of swelling of the soft tissues of the cheek, and pain on palpation of the anterolateral wall of the maxillary sinus [9,10,11]. The leading role in the diagnosis of odontogenic sinusitis still belongs to radiation methods of investigation [5,7,12,13,14]. Traditionally, this was done using X-ray in the naso-chin projection, plain radiographs of the skull in frontal and lateral projections. In the last two decades, there has been a widespread introduction of modern high-tech methods of radiation diagnostics—multispiral computed tomography and cone-beam computed tomography, which are significantly more informative [15,16,17,18,19,20]. The classic radiographic signs of sinus inflammation are thickened mucosa, darkening, and fluid levels. In a chronic process, there is a decrease in the transparency of the sinus [16,17,18,19].

Thus, it seems necessary to develop a method for the automated determination of the types of sinusitis, as well as to increase the reliability of differential diagnosis of various forms of odontogenic sinusitis.

## 2. Materials and Methods

For processing, we used tomographic sections in the axial projection parallel to the orbital–meatal line with a step of 1 mm and a spatial resolution of 0.4 mm. The initial data for the conducted researches are sets of images of tomographic sections received by means of the SOMATOM + spiral X-ray tomograph of SIEMENS firm (Munich, Germany). 

Our study considers 176 cases. All patients were divided into groups: the control group of 38 patients, with acute serous odontogenic sinusitis-35, acute purulent odontogenic sinusitis-36, chronic odontogenic sinusitis-38, and exacerbated chronic odontogenic sinusitis-32.

Examinations of patients were performed at the diagnostic center of the Kharkiv Regional Clinical Hospital, Head and Neck Surgery Department. Initially, the images are stored in DICOM format [10,11,21], converted using the standard utility DICOM_IMAGE in raster format BMP (Windows bitmap) with a size of 512 × 512 (x × y) and eight-bit representation of intensity levels. Preliminary processing of tomographic images was performed by the method of median filtration [22,23] to eliminate possible interference in the form of pulsed noise. We used methods of digital filtering, image segmentation and analysis, fluid dynamics, and statistical and discriminant analysis [24,25,26,27,28,29,30].

Mahalanobis distance is a measure of the distance between vectors of random variables, generalizing the concept of Euclidean distance, proposed by Indian statistician Mahalanobis in 1936. Using the Mahalanobis distance, you can determine the similarity of the unknown and known samples. It differs from Euclidean distance in that it takes into account correlations between variables and is scale invariant.

Rhinomanometry is a research method that makes it possible to objectively determine the function of nasal breathing. The method is very sensitive and is based on recording changes in air flow rate and intranasal pressure when breathing through the nose. We can use posterior rhinomanometry, first common for both halves of the nose, and then, with their alternate abturation, a comparative analysis of the obtained aerodynamic nasal drag coefficients was carried out.

## 3. Results

Most authors [31,32,33,34] diagnose odentogenic sinusitis mainly by computed tomography and external examination of the oral cavity. The paper proposes an automated approach to assessing tomographic data and additionally proposes to use a functional method for diagnosing nasal breathing disorders. This is based on the fact that inflammation in the sinus often causes inflammation or reactive edema in the nasal cavity, which leads to unilateral obstruction of nasal breathing (decreased nasal conduction).

Individual anatomical features of the paranasal sinuses and dentoalveolar system, the complexity of physiological and pathophysiological processes in this area, and the absence of actual standards of the norm and typical pathologies lead to the fact that differential diagnosis and assessment of the severity of the course of odontogenic sinusitis significantly depend on the measurement methods of significant indicators and have significant variability. Therefore, an urgent task is to expand the diagnostic capabilities of existing research methods, study the significance of the measured indicators, and substantiate the expediency of their use in the diagnosis of specific pathologies in an automated mode. This approach is especially relevant with the widespread introduction of objective instrumental diagnostic methods according to the criteria of evidence-based medicine. It is also necessary when developing new diagnostic methods, planning tools for surgical operations, and comparing the discriminant characteristics of the proposed method with existing ones. An important task in this case is the choice of informative parameters of diagnostics and control, as well as criteria by which the discriminant capabilities of the methods will be compared. When developing systems for automated analysis of medical data, it is advisable to introduce a two-level system for assessing diagnostic indicators: preliminary, which allows preliminary diagnostics (control, or differential diagnosis), and final, which allows, according to selected diagnostic indicators, obtaining the most reliable information about the state of the research object.

Modification of the method for diagnosis: It is necessary to carry out studies using spiral or cone-beam tomography and rhinomanometry. Without a rhinomanometric study, the reliability of the diagnosis is slightly reduced. Nevertheless, functional diagnostics of nasal breathing makes it possible to clarify morphological changes in the intranasal structures and paranasal sinuses, obtained from computed tomography data.

Particular attention should be paid to unilateral nasal breathing difficulties (from the side of the compromised paranasal sinus).

### 3.1. Possibilities of Preliminary Diagnosis of Odontogenic Sinusitis Based on Densitometric Analysis

To determine the radiological signs of odontogenic sinusitis, it is advisable to perform densitometric analysis in an automated mode, which is based on the fact that image intensity is determined by studying the value of image intensity (density) at each point along a certain trajectory, usually along a straight line. Intensity values in relative units (taking into account the choice of tomographic imaging window when displaying the range of the Hu scale of soft tissues in this case) are plotted on the ordinate axis, along the abscissa, indicating the coordinates of the points to be analyzed. This graph of intensity distribution along a certain direction is also called the brightness profile, or densitogram. The densitogram is used for densitographic analysis (analysis of the intensity distribution of the image along a certain direction), which is effective in the study of the density distribution of anatomical structures.

The construction of the densitogram is based on the choice of the initial Ts (x_s_, y_s_, k) and end Te (x_e_, y_e_, k) points of the trajectory on the corresponding tomographic section (k)-direct, or obtained by means of multiplanar reconstruction. Given that the measurements take place in the plane of one slice with a fixed number k, it is possible to consider only up to two dimensional coordinates of the start and end points Ts (x_s_, y_s_) and Te (x_e_, y_e_), respectively. The parametric equation of the line passing through these points is given as follows:

(1) $$\begin{array}{l} x(t)=x_{s}+(x_{e}-x_{s})\cdot t;\\ y(t)=y_{s}+(y_{e}-y_{s})\cdot t, \end{array}$$

where t∈[0,1] the choice of the step Δt of the change of the parameter t is determined, taking into account the distance d between the points Ts (x_s_, y_s_) and Te (x_e_, y_e_)

(2) $$d=\sqrt{{\left(x_{e}-x_{s}\right)}^{2}+{\left(y_{e}-y_{s}\right)}^{2}}$$

According to the formula

(3) $$\Delta t=\frac{1}{d}.$$

**Figure 1:**
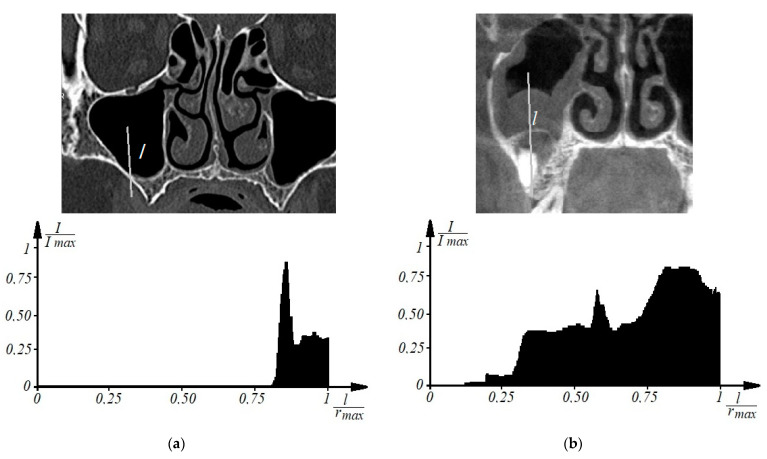
Examples of densitographic analysis according to spiral computed tomography: normal (**a**) and in the presence of a cyst of odontogenic origin in the maxillary sinus (**b**) (above is a tomographic section in coronary projection with the trajectory of the densitogram).

**Figure 2:**
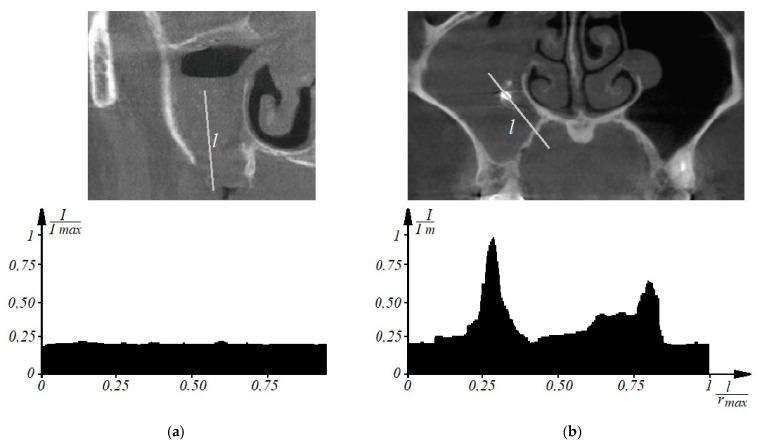
This is a figure. Schemes follow the same formatting. If there are multiple panels, they should be listed as: (**a**) Description of what is contained in the first panel; (**b**) Description of what is contained in the second panel. Figures should be placed in the main text near to the first time they are cited. A caption on a single line should be centered.

Thus, to determine the signs of odontogenic sinusitis in an automated mode, the patient undergoes a spiral computed tomography examination to diagnose the condition of the upper respiratory tract and paranasal sinuses. Images of axial spiral computed tomography sections of the upper jaw and maxillary sinus are obtained. In addition, multiplane and three-dimensional surface reconstructions of the studied area in the mode of reflection of bone structures are performed, the anatomical structure of the upper respiratory tract, the presence of deformations, displacements of bone formations, parameters of cranial defects, assessment of soft tissues and bone and bone sinuses are studied, and diagnostic decisions are made. Further, according to tomographic examination on tomographic images of the maxillary sinuses, the procedure of determining the center of the maxillary sinus involves construction of densitograms from the center of the maxillary sinus along radial trajectories in the lower hemisphere and analysis of the shape of densitometric data with characteristic features corresponding to typical pathologies. Normally (see Figure 1a), a typical densitogram appears with a minimum of intensity throughout the air cavity of the sinus and a pronounced peak of intensity at the bone border. In the presence of a cyst of odontogenic origin (see Figure 1b), the tissue content of the sinus and the additional border of the cyst are clearly visualized on the densitogram. In the presence of perforation of odontogenic origin in the maxillary sinus (see Figure 2a), the densitogram clearly visualizes the tissue content of the sinus and the absence of peak intensity at the missing border of the sinus. In the presence of a foreign body in the maxillary sinus (see Figure 2b), the densitogram on the background of the tissue content of the sinus clearly visualizes an additional area of high intensity, which corresponds to the high intensity of the foreign body.

Thus, due to the introduction of densitometric analysis of tomographic images of the maxillary sinuses at the previous level, it is possible to determine the characteristic densitometric features of various forms of odontogenic maxillary sinusitis and increase the efficiency of diagnosing pathologies of the paranasal sinuses, which is implemented in an automated mode.

### 3.2. Selection and Analysis of Diagnostic Indicators for Automated Diagnosis of Various Forms of Odontogenic Sinusitis

The effectiveness of solving problems of monitoring the states of objects with random properties, as a rule, depends on the correct choice of the most informative system of parameters (features) that are sensitive to changes in the characteristics of the object. Any control formally implements a testing procedure, the effectiveness of the result of which is determined by reliability—the probability of making the right decision [33]. This approach is complicated by the fact that when the properties of the research object are uncertain, the problem of selecting informative parameters becomes problematic. Especially if the metrological support of information transformations in the structure of the control system is difficult, which is often the case when considering the problems of medical diagnostics.

The choice of the optimal (according to the criterion of maximum reliability) system of information signs is a classical problem of statistical synthesis in conditions of a priori uncertainty [33]. The ranking of the signs in terms of information content is carried out in this case according to the value of the control reliability indicator or the probability of errors.

Let us consider the assessment of the possibility of using criteria and models of parametric recognition (discrimination) when comparing the diagnostic capabilities of X-ray cone-beam computed tomography in the diagnosis of various forms of odontogenic sinusitis.

Consider a linear discrimination model. The informative parameter X, used to obtain information about the a priori undefined properties of the control object, can be considered as a random variable. The latter, in the case of two states of an object ($\Theta_{0}$-norm, $\Theta_{1}$-deviation from the norm), is characterized by conditional probability distribution densities

(4) $$\begin{array}{l} X\approx f(X/\Theta_{0}){,}_{}if\Theta \in \Theta_{0},\\ X\approx f(X/\Theta_{1}){,}_{}if\Theta \in \Theta_{1}. \end{array}$$

If $m^{\left(0\right)},m^{\left(1\right)},\sigma^{{\left(0\right)}^{2}},\sigma^{{\left(1\right)}^{2}}$ are the means and variances of X for the conditions $\Theta \in \Theta_{0}$, and $\Theta \in \Theta_{1}$, accordingly, for normal (Gaussian) distributions $f(x/\Theta_{0})$, $f(x/\Theta_{1})$ the probability of a decision error in the form of object states is determined for variances $\sigma^{{\left(0\right)}^{2}}=\sigma^{{\left(1\right)}^{2}}$ through the probability integral Φ(⋅) [33]

(5) $$p_{\mathrm{er}}=1-\Phi (\delta /2)$$

where

(6) $$\delta =\left|\frac{m^{\left(0\right)}-m^{\left(1\right)}}{\sigma }\right|$$

The mean and standard deviation values included in Equation (6), respectively, are determined by the formulas below

$$\begin{array}{c} m=\frac{1}{n}\sum_{i=1}^{n}x_{i}\\ \sigma =\sqrt{\frac{1}{n}\sum_{i=1}^{n}{\left(x_{i}-m_{i}\right)}^{2}} \end{array}$$

where n is the number of measurements of the indicator under study.

If $\sigma^{{\left(0\right)}^{2}}\neq \sigma^{{\left(1\right)}^{2}}$, then the boundary for $p_{\mathrm{er}}$ can be estimated by the inequality

(7) $$p_{\mathrm{er}}\leq 1-\Phi (\delta /2)$$

In case of multiparameter control, when the number of informative parameters $x_{1},\ldots ,x_{n}$ is more than one (n≥2), the variable δ in expression (5) is described by the equation

(8) $$\delta =\sqrt{\sum_{i=1}^{n}{\left(\frac{m_{i}{}^{\left(0\right)}-m_{i}{}^{\left(1\right)}}{\sigma_{i}}\right)}^{2}}$$

where $\sigma_{i}$ is the standard deviation of the i-th indicator, which is determined by the formula

(9) $$\sigma_{i}=\max(\sigma_{i}{}^{\left(0\right)},\sigma_{i}{}^{\left(1\right)})$$

The square δ of this quantity from Equation (8)

(10) $$\delta^{2}=\sum_{i=1}^{n}{\left(\frac{m_{i}{}^{\left(0\right)}-m_{i}{}^{\left(1\right)}}{\sigma_{i}}\right)}^{2}$$

is called the quadratic normalized Euclidean distance between the controlled states (between the vectors of the state averages $\Theta_{0}$ and $\Theta_{1}$) [33].

The control object in this case is a vector-column of measured values

(11) $$\bar{x}=\left|\begin{array}{c} x_{1}\\ x_{2}\\ .\\ .\\ .\\ x_{n} \end{array}\right|$$

with conditional n-dimensional normal distribution density

Expression (10) assumes the mutual independence of the components of the vector with a linear model of discrimination [33,35,36,37].

The higher the error probability, the smaller, that is, the larger δ the square of the distance between the mean vectors normalized by variance.

Thus, the variables δ (or $\delta^{2}$) according to Equations (8) and (10) make it possible to quantitatively compare in terms of discriminating ability (in fact, in terms of information content) not only single informative diagnostic indicators, but also subsets (systems) of indicators.

At the same time, for each group of patients, statistical indicators were found: mean values $m_{i}^{\left(0\right)}$ and $m_{i}^{\left(1\right)}$ and standard deviations of the corresponding indicators, and for the calculation by Formula (8), the maximum standard deviation was selected according to Formula (9).

In calculations to determine the diagnostic significance of the parameters of X-ray cone-beam computed tomography in the diagnosis of various forms of odontogenic sinusitis, five informative parameters $x_{i}$($i=\bar{1,5}$) were involved, which are displayed in ascending order of numbering:

$x_{1}$—the density of the fluid content of the sinus, Hu;

$x_{2}$—the relative indicator of the area of the opening of the anastomosis,%;

$x_{3}$—the relative indicator of the volume of the mucous membrane of the sinus,%;

$x_{4}$—the relative indicator of the volume of the fluid content of the sinus,%,

$x_{5}$—coefficient of aerodynamic nose resistance A, kPa/(L/s).

The first four indicators were directly measured on tomograms, and the coefficient of aerodynamic nose drag was also determined indirectly from tomographic data according to the methodology given in the literature [32,38,39]. Let us consider in detail the method of their determination.

For the first diagnostic indicator, the density of the liquid content was determined by measuring the average X-ray absorption in tissues, determined by the Hounsfield scale (in units of Hu, respectively). This is an important diagnostic criterion, since air normally has large negative values on the Hounsfield scale; also, the density of serous contents is significantly less than the purulent contents of the sinuses, which is significant in the differential diagnosis of acute forms of odontogenic sinusitis, respectively.

**Figure 3:**
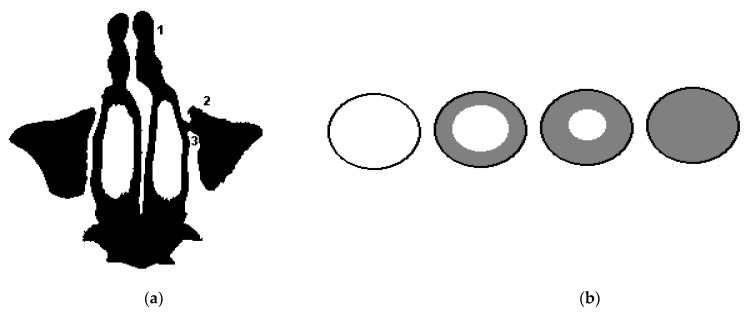
Segmentation of the airways of the nasal cavity according to tomographic data and a schematic illustration of the overlap of the natural anastomosis of the maxillary sinus: (**a**) segmentation of the airways of the nasal cavity according to tomographic data in the axial projection at the level of the middle nasal passage: 1—airways, 2—maxillary sinus, 3—natural anastomosis of the maxillary sinus sinuses (for the left side of the nasal cavity); (**b**) a schematic illustration of the overlap of the anastomosis of the maxillary sinus: from left to right, with a working (unblocked) anastomosis, 60% free, 30% free, and with completely blocked anastomosis.

The second diagnostic indicator is the indicator of the opening of the natural anastomosis of the maxillary sinus, which goes into the middle nasal passage, showing (in percentage) how much the anastomosis is free for the process of physiological aeration and ensuring mucociliary clearance. Figure 3a shows the segmentation of the airways of the nasal cavity 1 according to tomographic data in the axial projection at the level of the middle nasal passage with the designation of the maxillary sinus 2 and natural fistula 3 (designations are given for the left side of the nasal cavity). Figure 3b shows a schematic designation of the sagittal section of the natural anastomosis of the maxillary sinus, from left to right, with a working (unblocked) anastomosis, 60% free, 30% free, and with a completely blocked anastomosis.

**Figure 4:**
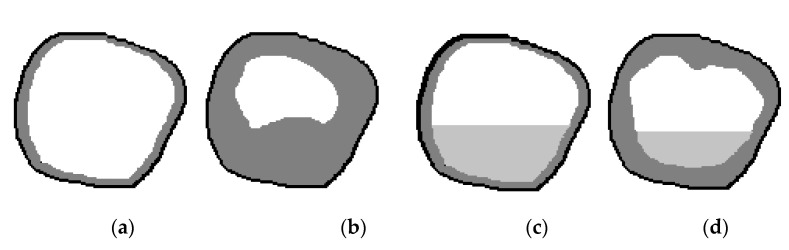
Schematic illustration of the maxillary sinus in a sagittal projection: (**a**) at a conditional rate, (**b**) at 60% filled sinus cavity with polyposis content; (**c**) when filling the sinus by 40% liquid content; (**d**) filling of the sinus with liquid and polyposis contents by 30% and 20%, respectively.

Figure 4 shows schematic illustrations of the maxillary sinus in a sagittal projection to explain the third and fourth diagnostic indicators (the relative indicator of the volume of the sinus mucosa, %, and the relative indicator of the volume of the fluid content of the sinus, %): a—at a conditional rate; b—at 60% filled sinus cavity with polypous contents, characteristic of chronic odontogenic sinusitis; c—when the sinus is filled by 40% with liquid contents, characteristic of a serous or purulent process; d—filling the sinus with polyposis contents (by 30%) and liquid (by 20%), characteristic of exacerbated chronic odontogenic sinusitis. Examples of real tomographic images with different contents of the sinuses are shown earlier in Figure 1 and Figure 2.

**Figure 5:**
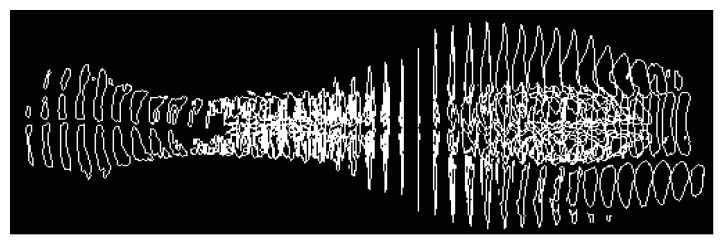
Illustration of a set of frontal sections of the air channel of the nasal cavity in a sagittal projection obtained as a result of segmentation of tomographic data.

The index of changes in nasal breathing (aerodynamic nasal drag coefficient) can be determined from a set of sections of the air channel of the nasal cavity obtained from tomographic data, an example of which is shown in Figure 5 [38], according to the methodology given in [32,38,39]. The essence of this approach is to calculate the resistance to air flow (during breathing) of the nasal cavity, represented as a channel with a complex configuration [32,38] with the construction of segmented geometric models based on tomographic data [32,38,39,40].

When calculating pressure losses in complex pipelines, which include parallel channels of the nasal passages, the air flow through each of them is equal to the total, based on the continuity equation according to [32]

(12) $$Q_{\sum }=Q_{1}+Q_{2}$$

The pressure loss in each channel is determined based on the constancy of the pressure difference between the common inlet (atmospheric pressure) and the outlet to the nasopharynx (where the nasal passages exit) according to

(13) $$\Delta p_{1}=\Delta p_{2}=\Delta p=\mathrm{const}$$

therefore, the pressure loss can be expressed as

(14) $$\Delta p_{1}=\Delta p_{l,1}+\Delta p_{L.R,1}=\sum \lambda_{1}\rho_{1}\frac{\Delta l_{1}}{d_{r}}\frac{Q_{1}^{2}}{2S_{1}^{2}}+\sum \xi_{1}\rho_{1}\frac{Q_{1}^{2}}{2S_{1}^{2}}=Q_{1}^{2}A_{1}$$

(15) $$\Delta p_{2}=\Delta p_{l}{}_{,2}+\Delta p_{L.R}{}_{,2}=\sum \lambda_{2}\rho_{2}\frac{\Delta l_{2}}{d_{r}}\frac{Q_{2}^{2}}{2S_{2}^{2}}+\sum \xi_{2}\rho_{2}\frac{Q_{2}^{2}}{2S_{2}^{2}}=Q_{2}^{2}A_{2}$$

where $\Delta p_{l}$ and $\Delta p_{L.R}$ are the pressure losses along the length and on local resistances for the corresponding sections of each channel,

Δl—the length of the channel or calculated section, m,

S—the area of the calculated channel section, m,

$d_{h}$—hydraulic (equivalent) diameter [39],

ρ—air density, = 1.205 kg/m^3^,

ξ—dimensionless coefficient of local hydraulic losses,

λ—dimensionless coefficient of hydraulic friction (Darcy coefficient), equal λ=64/Re for laminar, and λ=0,32/Re0,25 turbulent air flow modes, respectively [32],

A_1_ and A_2_ are constant values for the aerodynamic drag of the nasal passages, determined from Formulas (14) and (15) as

(16) $$A_{1}=\sum \lambda_{1}\rho_{1}\frac{\Delta l_{1}}{d_{r}}\frac{1}{2s_{1}^{2}}+\sum \xi_{1}\rho_{1}\frac{1}{2s_{1}^{2}}$$

(17) $$A_{2}=\sum \lambda_{2}\rho_{2}\frac{\Delta l_{2}}{d_{r}}\frac{1}{2s_{2}^{2}}+\sum \xi_{2}\rho_{2}\frac{1}{2s_{2}^{2}}$$

Taking into account the above Formulas (16) and (17), for the laminar and turbulent regime, the pressure drops are determined according to the formulas

(18) $$\begin{array}{c} \Delta p_{1}=\Delta p_{2}=\Delta p=Q_{1}^{}\cdot A_{1}^{\left(\Lambda \right)}=Q_{2}^{}\cdot A_{2}^{\left(L\right)}\\ \Delta p_{1}=\Delta p_{2}=\Delta p=Q_{1}^{2}\cdot A_{1}^{\left(T\right)}=Q_{2}^{2}\cdot A_{2}^{\left(T\right)} \end{array}$$

Experimental verification of these data can be carried out according to the data of anterior or posterior active rhinomanometry [32,38], taking into account the breathing mode and individual physiological variability, which correspond to the pressure drop across the nasal cavity and the corresponding air flow rate.

## 4. Discussion

In the calculations, the indicators associated with geometric measurements were taken relatively in order to maximally exclude the influence of individual anatomical variability.

The calculated data on the indicators of nasal resistance can be compared with those directly obtained using the methods of posterior or anterior active rhinomanometry, in which the pressure drop Δp across the nasal cavity and the air flow Q caused by it during breathing are directly measured. Based on the data obtained, the coefficient of aerodynamic nose drag is calculated

(19) $$A=\frac{\Delta p}{Q}$$

which will correspond to either the integral index of aerodynamic nose drag or the corresponding nasal passage (according to Formulas (16) and (17)), depending on the measurement method. Thus, it is possible to obtain an experimental assessment of the aerodynamic parameters of the nasal cavity, however using an additional functional research method, which, given the low prevalence of rhinomanometric equipment in clinics, is not always possible. Therefore, the determination of the functional parameters of nasal breathing according to the geometric characteristics of the nasal cavity based on tomographic data with an error of no more than 20% [32] is quite acceptable without using additional research methods. The disadvantage of this method is a rather large computational complexity associated with the segmentation of the anatomical structures of the nasal cavity and the construction of an aerodynamic model of the upper respiratory tract.

The patients were divided into the corresponding groups: with acute serous odontogenic sinusitis, acute purulent odontogenic sinusitis, chronic odontogenic sinusitis, exacerbated chronic odontogenic sinusitis, and the control group, which is a conditional norm. Indicators are not ranked according to the contribution of information content to the discrimination model [30,41,42,43] for the consistency of the illustrative material and convenience when comparing values.

The results of discriminant analysis when comparing the main tomographic indicators for these pathologies with the norm are given in Table 1 and Table 2.

**Figure 6:**
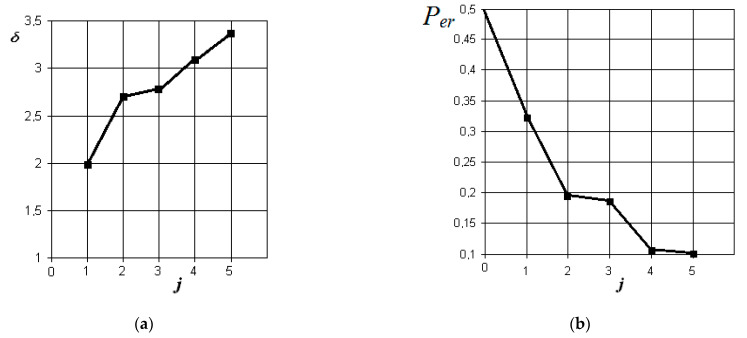
The results of discriminant data analysis when comparing the norm with acute serous odontogenic sinusitis: (**a**) the dependence of the increase in the normalized Euclidean distance as signs are added to the discrimination model δ=f(j); (**b**) the dependence of the decrease in the probability of a decision error as signs are added to the discrimination model $p_{\mathrm{er}}^{}=f(j)$: j = 5 is the dimension of the space of informative parameters.

**Figure 7:**
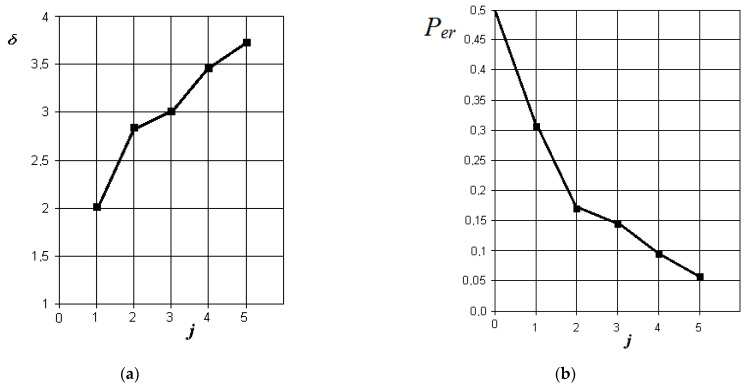
The results of discriminant data analysis when comparing the norm with acute purulent odontogenic sinusitis: (**a**) the dependence of the increase in the normalized Euclidean distance as signs are added to the discrimination model δ=f(j); (**b**) the dependence of the decrease in the probability of a decision error as signs are added to the discrimination model $p_{\mathrm{er}}^{}=f(j)$: j = 5-is the dimension of the space of informative parameters.

**Figure 8:**
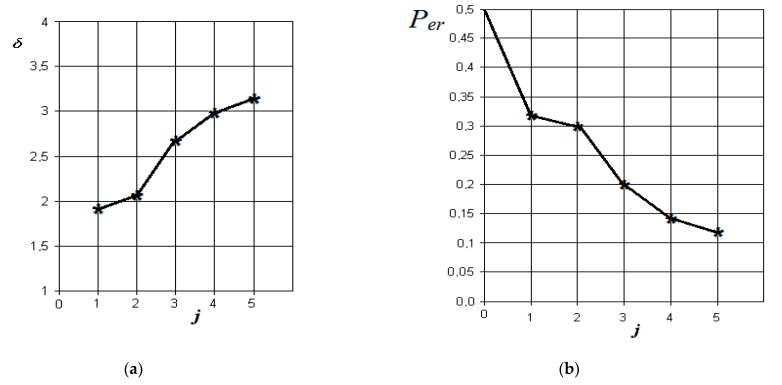
Results of discriminant analysis of data when comparing the norm with chronic odontogenic sinusitis: (**a**) dependence of the increase in the normalized Euclidean distance as signs are added to the discrimination model δ=f(j); (**b**) -the dependence of the decrease in the probability of error in decision-making as features are added to the discrimination model $p_{\mathrm{er}}^{}=f(j)$: j = 5-is the dimension of the space of informative parameters.

**Figure 9:**
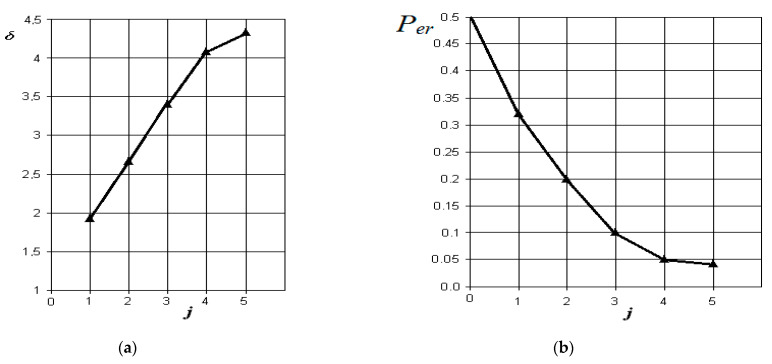
The results of discriminant data analysis when comparing the norm with exacerbated chronic odontogenic sinusitis: (**a**) the dependence of the increase in the normalized Euclidean distance as signs are added to the discrimination model δ=f(j); (**b**) the dependence of the decrease in the probability of decision-making errors as features are added to the discrimination model $p_{\mathrm{er}}^{}=f(j)$: j = 5-is the dimension of the space of informative parameters.

The graphs of the increase in the normalized Euclidean distance and the change in the probability of an error in making a diagnostic decision when monitoring various pathologies with the norm are shown in Figure 6, Figure 7, Figure 8 and Figure 9, respectively.

From the analysis of the graphs in Figure 6, it is obvious that in acute serous odontogenic sinusitis, the most significant indicators are the presence of fluid content and its X-ray density in the sinus. Indicators associated with impaired nasal breathing, overlapping of the natural anastomosis, and thickening of the mucous membrane of the maxillary sinus are significantly less significant in this pathology. In this case, the resulting probability of a control error is 0.1.

From the analysis of the graphs in Figure 7, it is obvious that in acute purulent odontogenic sinusitis, the indicators of the presence of liquid contents and its X-ray density in the sinus are also the most significant. However, to them is essentially added an indicator associated with the overlap of the area of the natural sinus fistula. Violation of nasal breathing also begins to affect. The indicator associated with the possibility of thickening of the mucous membrane of the maxillary sinus is significantly less significant in this pathology. In this case, the resulting probability of a control error is about 0.05.

From the analysis of the graphs in Figure 8, it is obvious that in chronic odontogenic sinusitis, the most significant indicators are those associated with the presence of fluid content and its radiological density in the sinus. However, to them, the indicator associated with thickening of the mucous membrane of the maxillary sinus is significantly added. Less significant in this form are the indicator of the overlap of the natural sinus fistula and disturbance of nasal breathing, which is possibly associated with the compensatory mechanisms of the body during a long period of the disease. In this case, the resulting probability of a control error is about 0.12.

From the analysis of the graphs in Figure 9, it is obvious that with exacerbated chronic odontogenic sinusitis, the most significant indicators are those associated with the presence of fluid content and its radiological density in the sinus, as well as the overlap of the natural fistula and thickening of the sinus mucosa. The role of changes in nasal breathing does not add significantly greater significance to the process of diagnosing this form. In this case, the resulting probability of control error is the least (in comparison with other forms) and is about 0.04.

The greatest reliability of control with the norm is achieved in the diagnosis of exacerbated chronic and acute purulent odontogenic sinusitis (the probabilities of a decision error are close small values of 0.04 and 0.05, respectively), which is associated with pronounced clinical manifestations of these pathologies in comparison with the norm.

Table 3, Table 4, Table 5 and Table 6 below show the results of differential diagnosis of the above forms of odontogenic sinusitis.

**Figure 10:**
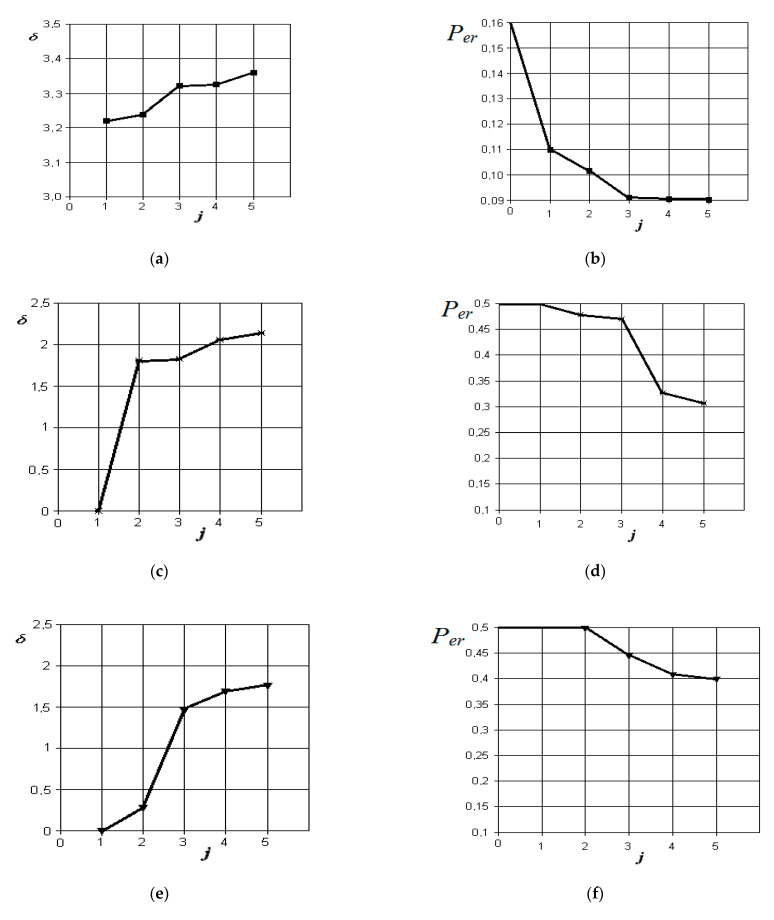

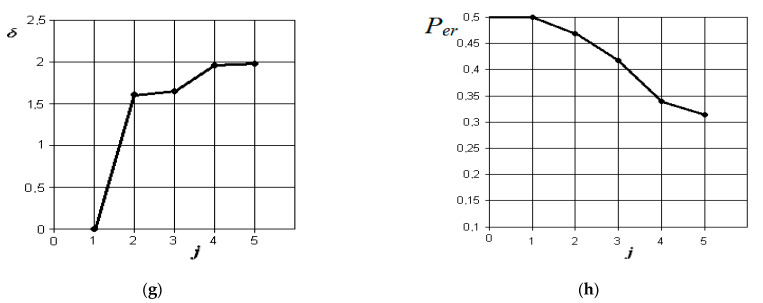
Results of discriminant analysis of data in the differential diagnosis of forms of acute serous and purulent odontogenic sinusitis (**a**,**b**), forms of chronic and acute purulent odontogenic sinusitis (**c**,**d**), forms of acute purulent and exacerbated chronic odontogenic sinusitis (**e**,**f**), and forms of chronic and exacerbated chronic odontogenic sinusitis (**g**,**h**): first column—the dependence of the increase in the normalized Euclidean distance as features are added to the discrimination model δ=f(j); second column—the dependence of the decrease in the probability of error in decision-making as features are added to the discrimination model $p_{\mathrm{er}}^{}=f(j)$: j = 5 is the dimension of the space of informative parameters.

The corresponding graphs of differential diagnosis of the studied forms of odontogenic sinusitis are shown in Figure 10.

From the analysis of the graphs in Figure 10, it is obvious that when comparing the forms of acute serous and purulent odontogenic sinusitis, the most significant indicators are those that characterize the differences in the density of the fluid content in the sinus, overlap of the natural fistula area, and thickening of the mucous membrane of the maxillary sinus. Indicators associated with the difference in the volume of the contents in the maxillary sinus and the violation of nasal breathing are significantly less significant in this pathology. The resulting probability of a control error is 0.09.

From the analysis of the graphs in Figure 10c,d, it is obvious that when comparing the forms of chronic and acute purulent odontogenic sinusitis, the most significant indicator is the one that characterizes the difference in the presence of fluid content in the maxillary sinus. Further in importance are the indicators of overlap of the natural anastomosis of the maxillary sinus, changes in nasal breathing, and thickening of the mucous membrane of the maxillary sinus. The resulting probability of a control error is 0.31.

From the analysis of the graphs in Figure 10e,f, it is obvious that when comparing the forms of acute purulent and exacerbated chronic odontogenic sinusitis, the most significant indicator is the one that characterizes the difference in the thickening of the mucous membrane of the maxillary sinus. Other indicators, such as the difference in the presence of fluid content, overlap of the area of the natural anastomosis of the maxillary sinus, and changes in nasal breathing, do not significantly affect the improvement of the discrimination model. The resulting probability of a control error is 0.4.

From the analysis of the graphs in Figure 10g,h, it is obvious that when comparing the forms of chronic and exacerbated chronic odontogenic sinusitis, the most significant indicators are those that characterize the differences in the overlap of the natural fistula area and thickening of the maxillary sinus mucosa. Other indicators, such as the presence of fluid content and changes in nasal breathing, do not significantly affect the improvement of the discrimination pattern. The resulting probability of a control error is 0.32.

## 5. Conclusions

It is necessary to repeat the nasal breathing test several times at intervals of at least 2 h to exclude the influence of the nasal cycle on the result.

Disadvantages: It is necessary to have sophisticated medical technology available for both imaging and functional diagnostics of nasal breathing. Funk diagnostic methods have a relatively low repeatability of results (reproducibility), which are influenced by various factors, as well as concomitant inflammatory diseases of the nasal cavity, past injuries, developmental anomalies, atrophic, vasomotor rhinitis, etc.

When analyzing tomographic data, in some cases, it is not possible to determine the center of the paranasal sinus on the tomographic section, which leads to the placement of markers in automatic mode. The window for soft tissue imaging must be carefully selected.

The measurement error may additionally be associated with the peculiarity of performing multiplanar reconstructions when carrying out densitometry based on tomographic data, as well as the peculiarities of the individual structure of the paranasal sinuses, the presence of additional cells, etc., which must be taken into account by a specialist in advance. The assessment of the clinical result is based on a comprehensive study in accordance with the current protocols, and the proposed method allows you to add additional information and evidence to the obtained medical data. A functional study of nasal breathing makes it possible to assess the influence of the inflammatory process occurring in the sinus on the structures of the nasal cavity by changing nasal conductivity.

From the analysis of the data obtained, it follows that in the differential diagnosis of various forms of odontogenic sinusitis, the greatest difference in indicators and, as a consequence, the least probability of making a diagnostic decision is observed when comparing the forms of acute serous and purulent odontogenic sinusitis (0.09), which is associated with a significant the difference in the density of the fluid content of the maxillary sinus and makes the discrimination model more precise. In other cases, when making a differential diagnosis, the density of the fluid contents of the maxillary sinus is almost identical and cannot be criteria for making a differential diagnosis. The lowest reliability of differential diagnosis (probability of error 0.4) is achieved when comparing indicators of forms of acute purulent and exacerbated chronic odontogenic sinusitis and is due to the fact that only the indicator characterizing the difference in thickening of the mucous membrane of the maxillary sinus is significant in the analysis of tomographic data. When comparing other forms of odontogenic sinusitis, the probability of discrimination error is about 0.3. At the same time, it should be noted that it is a quite logical conclusion that the reliability of differential diagnosis of forms of odontogenic sinusitis was significantly lower than the reliability of comparison (control) with the norm. This is due to the proximity of the values of the measured indicators in pathological conditions compared with the norm.

Preliminary differential diagnosis of odontogenic sinusitis can be performed by densitometric analysis of tomographic images of the maxillary sinuses, performed by frontal multiplanar reconstructions according to a given algorithm. The presence of characteristic changes in the densitography of the maxillary sinus allows the determination of the relevant pathological processes and increases the efficiency of diagnosis of pathologies of the paranasal sinuses, which is implemented in an automated mode.

When a new patient appears for him, the above indicators are calculated for him from the set of tomographic data, and the normalized Euclidean distances to the center of the clusters corresponding to the studied pathologies are calculated. The shortest distance to the center of one of the clusters will correspond to the most probable form of odontogenic sinusitis. Additionally, these distances can be ranked and determine the probabilities of compliance of the case with specific pathologies.

The development of a system of automated diagnosis of odontogenic sinusitis will allow novice specialists to obtain an approximate diagnosis based on the input data, which can be useful in obtaining diagnostic skills.

The prospect of the work is the analysis of diagnostic errors, the substantiation of the main medical and technical characteristics, and practical recommendations for the system for the diagnosis of odontogenic sinusitis and subsequent clinical trials of the completed development.

## Figures and Tables

**Table 1:** Comparison (control) of the norm with acute serous odontogenic sinusitis and acute purulent odontogenic sinusitis.

Pathology	Conditional Norm		Acute Serous Odontogenic Sinusitis		Acute Purulent Odontogenic Sinusitis	
Index/Parameter	ε	σ_ε_	ε	σ_ε_	ε	σ_ε_
Density of fluid content of the sinus, Hu	−630	330	19	4.3	37	6.2
Relative indicator of the opening area of the anastomosis,%	96	39	24	12	20	9
Relative index of the volume of the sinus mucosa, %	10	5.7	16.2	14.5	27	16
The relative indicator of the volume of fluid content of the sinus, %	0	0	52	30.5	54.3	32
Aerodynamic nose drag coefficient A, kPa/(L/s)	0.45	0.24	1.58	0.87	2.2	1.12
δ	3.34				3.78	
$p_{\mathrm{error}}$	0.1				0.06	

**Table 2:** Comparison (control) of the norm with chronic odontogenic sinusitis and exacerbated chronic odontogenic sinusitis.

Method Type	Conditional Norm		Chronic Odontogenic Sinusitis		Acute Chronic Odontogenic Sinusitis	
Index/Parameter	ε	σ_ε_	ε	σ_ε_	ε	σ_ε_
Density of fluid contents of the sinus, Hu	−630	330	39	6.4	38.5	6.1
Relative indicator of the opening area of the anastomosis,%	96	39	64	25.5	23	11.2
The relative indicator of the volume of the mucous membrane of the sinus,%	10	5.7	62	25	65	31
The relative indicator of the volume of the fluid content of the sinus,%	0	0	15	9	28	12
Aerodynamic nose drag coefficient A, kPa/(L/s)	0.45	0.24	1.72	1.12	1.94	1.28
δ	3.16				4.29	
$p_{\mathrm{error}}$	0.12				0.04	

**Table 3:** Main indicators and results of differential diagnosis of forms of acute serous and purulent odontogenic sinusitis.

Pathology	Acute Serous Odontogenic Sinusitis		Acute Purulent Odontogenic Sinusitis	
Index/Parameter	ε	σ_ε_	ε	σ_ε_
Density of fluid contents of the sinus, Hu	19	4.3	37	6.2
Relative indicator of the opening area of the anastomosis,%	24	12	20	9
The relative indicator of the volume of the mucous membrane of the sinus,%	16.2	14.5	27	16
The relative indicator of the volume of the fluid content of the sinus,%	52	30.5	54.3	32
Aerodynamic nose drag coefficient A, kPa/(L/s)	1.58	0.87	2.2	1.12
δ	3.36			
$p_{\mathrm{error}}$	0.09			

**Table 4:** Main indicators and results of differential diagnosis of forms of chronic and acute purulent odontogenic sinusitis.

Pathology	Chronic Odontogenic Sinusitis		Acute Purulent Odontogenic Sinusitis	
Index/Parameter	ε	σ_ε_	ε	σ_ε_
Density of fluid contents of the sinus, Hu	399	6.4	37	6.2
Relative indicator of the opening area of the anastomosis,%	24	64	25.5	9
The relative indicator of the volume of the mucous membrane of the sinus,%	62	25	27	16
The relative indicator of the volume of the fluid content of the sinus,%	15	9	54.3	32
Aerodynamic nose drag coefficient A, kPa/(L/s)	1.72	1.12	2.2	1.12
δ	2.18			
$p_{\mathrm{error}}$	0.31			

**Table 5:** Main indicators and results of differential diagnosis of forms of acute purulent and exacerbated chronic odontogenic sinusitis.

Pathology	Acute Purulent Odontogenic Sinusitis		Acute Chronic Odontogenic Sinusitis	
Index/Parameter	ε	σ_ε_	ε	σ_ε_
Density of fluid contents of the sinus, Hu	37	6.2	38.5	6.1
Relative indicator of the opening area of the anastomosis,%	64	25.5	23	11.2
The relative indicator of the volume of the mucous membrane of the sinus,%	27	16	65	31
The relative indicator of the volume of the fluid content of the sinus,%	54.3	32	28	12
Aerodynamic nose drag coefficient A, kPa/(L/s)	2.2	1.12	1.94	1.28
δ	1.71			
$p_{\mathrm{error}}$	0.4			

**Table 6:** Main indicators and results of differential diagnosis of forms of chronic and exacerbated chronic odontogenic sinusitis.

Pathology	Chronic Odontogenic Sinusitis		Acute Chronic Odontogenic Sinusitis	
Index/Parameter	ε	σ_ε_	ε	σ_ε_
Density of fluid contents of the sinus, Hu	39	6.4	38.5	6.1
Relative indicator of the opening area of the anastomosis,%	64	25.5	23	11.2
The relative indicator of the volume of the mucous membrane of the sinus,%	62	25	65	31
The relative indicator of the volume of the fluid content of the sinus,%	15	9	28	12
Aerodynamic nose drag coefficient A, kPa/(L/s)	1.72	1.12	1.94	1.28
δ	1.98			
$p_{\mathrm{error}}$	0.32			

## Data Availability

The data were taken from the database of patients of the regional clinical hospital, the department of Otorhinolaryngology (Head and Neck Surgery), which is the clinical base for the department of Otorhinolaryngology of the Kharkov National Medical University.
